# Efficacy of low dose capecitabine and sorafenib in patients with advanced alfa-fetoprotein secreting hepatocellular carcinoma: a 1 year experience

**DOI:** 10.1186/s40064-016-3376-x

**Published:** 2016-09-29

**Authors:** Amr Shaaban Hanafy

**Affiliations:** Hepatogastroenterology Division, Internal Medicine Department, Zagazig University, 40-Mostafa Fouad St, Sharkia, Zagazig, 44519 Egypt

**Keywords:** Low dose, Capecitabine, Sorafenib, HCV, Hepatocellular carcinoma

## Abstract

**Background:**

There is no global consensus for the optimal management of HCC. Most of patients at the time of diagnosis are not candidate for potentially curative therapy. The study aimed to evaluate the efficacy of low dose capecitabine combined with sorafenib in subset of Egyptian HCV patients presented with advanced HCC unfit for surgical or locoregional therapies.

**Methods:**

15 patients with advanced HCC, unfit for surgical or locoregional intervention, with PS <2 recieved Capecitabine 500 mg/day with sorafeneb 200 mg twice daily till normalization of AFP then the treatment was modified to capecitabine 250 mg every other day and sorafenib 400 mg once daily. They were followed every 3 months for size, number of focal masses and AFP. 30 patients were selected as a control group, they received supportive therapy (n = 15) or sorafenib only (n = 15).

**Results:**

After 10 months of therapy, 6 patients showed complete response (40 %) with complete recanalization of portal vein (n = 2) and treatment was stopped and the others (n = 4) showed partial portal vein recanalization so, treatment is continued till now. 1 patient (6.7 %) showed recurrence of the disease and died after 1 month, 8 patients showed partial response (53.3 %) and still on treatment. The control groups showed a highly significant reduction in survival when compared to patients who received capecitabine and sorafenib (12.9 ± 2.1, 7.9 ± 0.9, 4.5 ± 1.3 months, p = 0.000).

**Conclusions:**

Combined low dose capecitabine and sorafenib proved to be safe with low toxicity profile and deserves further attention as a convenient, outpatient-based chemotherapy in patients with advanced HCC.

## Background

The continued appearance of new cases of hepatocellular carcinoma (HCC) with horribly increasing incidence making it the fifth common worldwide solid tumor, unfortunately, most of the cases present in an advanced or inoperable stage (Parkin et al. 1999).

In Egypt, the majority of patients are due to HCV, and actually, they present lately with reduced median survival and so therapy in most cases is palliative. The treatment options either curative in the form of hepatic resection or liver transplantation and palliative to include transcatheter arterial chemoembolization (TACE) which is associated with a partial response in 10–55 %, radioembolization, and medical therapy with sorafenib which is an oral multikinase inhibitor that had led to significant lengthening of short term-survival when compared to placebo (Lovet et al. 2008).

Alfa-fetoprotein (AFP) is a useful marker in patients with cirrhosis and focal hepatic masses. Values exceeding 400 ng/ml are highly suggestive of HCC. A persistently elevated AFP above normal value without abnormal radiological findings may predict the future development of HCC (Forner et al. 2009). About 50–70 % of advanced HCC secrete AFP (Peng et al. 2004).

There are no established guidelines for the adequate management of HCC, however according to Barcelona Clinic Liver Cancer (BCLC) staging system which is a widely endorsed system; patients with early HCC defined as a single nodule or three nodules <3 cm in diameter and a performance status (PS) score of 0 with the absence of extrahepatic seedling, are suitable for potentially curative therapies as surgical resection, orthotopic liver transplantation or interventional ablation by radiofrequency, microwave or ethanol injection.

Patients with adequate liver function and still asymptomatic (PS = 0) with large (>5 cm) or multifocal tumors without vascular or extrahepatic invasion can be treated with transarterial chemoembolization (TACE) (Lopez et al. 2006). Careful analysis of all randomized controlled trials showed that treatment with TACE is associated with a significantly higher 2-year survival rate than in untreated groups (Llovet et al. 2003).

Better results were achieved with the use of gelatin sponge particles and an emulsion of cisplatin with Lipiodol (Lo et al. 2002). The use of drug-eluting beads (DEBs) as doxorubicin-loaded beads rather than doxorubicin Lipiodol emulsion was associated with a better toxicity profile, antitumor activity (51.6 vs 43.5 %, respectively), and tolerability (Lammer et al. 2010) with a longer time to tumor progression than bland TACE with non-loaded beads (Malagari et al. 2010).

In neoplastic portal vein thrombosis (PVT), TACE is contraindicated, however radioembolization or selective internal radiation therapy (SIRT) comprising a catheter-based delivery of yttrium-90 (90Y)-embedded microspheres into the hepatic artery which selectively emit high-energy, low-penetration radiation to the tumor (Ibrahim et al. 2008).

A study 2015 compared both SIRTS vs DEBs in the management of advanced HCC and revealed that although a lower rate of tumor progression was noted in the SIRT group, it was nullified by a greater incidence of liver cell failure (Pitton et al. 2015).

Sorafenib is an inhibitor of tyrosine protein kinases (VEGFR and PDGFR) and Raf kinases (mainly C-Raf than B-Raf) (Keating and Santoro 2009), it induces autophagy which may interfere with tumor growth. However, drug resistance may arise which is of great importance (Zhang et al. 2014).

It showed a significant improvement in patients with advanced HCC when compared to placebo (hazard ratio 0.69; 95 % CI 0.55–0.87; p = 0.0001). Both median survival and time to progression showed a 3-month improvement. However, there was no difference in the quality of life or symptoms caused by progression of liver disease due to side effects of the large dose of sorafenib (Patt et al. 2004). It is suitable for Child-Pugh class A and B with close follow-up.

Capecitabine is a prodrug with complete absorption from gastrointestinal mucosa. It is converted to the active metabolite 5-fluorouracil by thymidine phosphorylase enzyme; the latter exists in higher levels in cancerous tissues and the liver compared with normal tissue rendering it more specific and it was found to be safe in patients with liver cirrhosis (Twelves et al. 1999).

The increasing number of patients presented with advanced HCC with no other eligible treatment options that can be offered for them as surgery, local ablative therapies or chemoembolization will put us in a challenging situation, so the aim of this work is to evaluate the efficacy of low dose capecitabine combined with sorafenib which are potentially safe and effective drugs based on previous studies, in subset of Egyptian HCV patients presented with advanced HCC who were unfit for surgical or loco regional therapies.

## Subjects and methods

In the period extending from April 2014 till February 2016, at Hepatology outpatient clinic—Zagazig University hospital, the efficacy of capecitabine combination with sorafenib was evaluated in the management of advanced inoperable HCC unfit for surgical or locoregional therapies, and the patients had an adequate hepatic reserve with PS ≤2.

From 60 patients presented with HCC, 15 male patients (25 %) were selected according to the predetermined inclusion criteria. The number of patients was determined according to diagnosed patients during this period of the study. They were treated after being informed about the possible side effects of medications and informed consent was obtained from them. All procedures performed were in accordance with the ethical standards of Zagazig university research committee and with the 1964 Helsinki declaration and its later amendments.

The control group was age and sex matched and divided into 2 subgroups; subgroup 1 which included 15 patients who received only supportive therapy and subgroup 2 which included 15 patients who received sorafenib only, they were followed up every 2 months.

### Inclusion criteria

- Hepatocellular carcinoma that is not suitable for surgical or loco-regional interventions with adequate bone marrow function denoted by average neutrophil count more than 1500/µl, Hemoglobin level more than 8.5 g/dl, Platelet count >60,000/µl. Serum creatinine <2.0 mg/dl, and creatinine clearance >30 ml/min calculated by Cockcroft–Gault equation. Adequate hepatic reserve (Patients with a Child-Pugh class A–B) with Bilirubin <2.8 mg/dl, INR <1.7, albumin ≥2.8 gm/dl, ALT and AST ≤three times upper limit of normal.
- Performance Status <2, i.e. asymptomatic and fully active, able to carry on all pre-disease activities or symptomatic but completely ambulatory.

### Exclusion criteria

- Any previous systemic therapy including chemotherapy.
- Uncontrolled ascites.
- Unstable cardiac diseases including Congestive heart failure > class II NYHA, unstable angina, new onset angina, myocardial infarction, or any uncontrolled cardiac troubles within the preceding 6 months as capecitabine may induce coronary vasospasm.
- Metastatic disease or any co-morbid state that increases the chance of complications of therapy.
- Thromboembolic events within the past 6 months, any hemorrhagic event within 2 months of therapy.

### Treatment schedule and follow up

Capecitabine was administered orally once daily at a dose of 500 mg per day together with sorafenib 200 mg twice daily till return of AFP to normal level then the treatment was modified to capecitabine 250 mg every other day and sorafenib 400 mg once daily until complete disappearance of the lesions with recanalization of the portal vein or non-response in the form of progression of disease, unbearable toxicity or worsening of liver function with progression of Child-Pugh class. The control group received sorafenib only in a dose of 400 mg twice daily or supportive treatment as silymarin 140 mg t.d.s, ursodeoxycholic acid 500 mg and symptomatic treatment for bleeding tendency and portal hypertension and abdominal pain.

The safety, tolerability and occurrence of side effects with the use of these agents and their effect on tumor size were evaluated according to response evaluation criteria in solid tumors. evaluation was by computerized tomography every 3 months and laboratory follow up including liver, kidney function tests, complete blood count and AFP every 2 weeks in the first 2 months then every month.

- Complete response means the disappearance of all target lesions.
- Partial response means more than 30 % decrease in the sum of the longest diameter of the tumorous lesions.
- Stable disease means neither sufficient decrease nor increase in the sum of the longest diameter of the lesions to classify them into a progressive or regressive category.
- Progressive disease denotes 20 % increase in the sum of the longest diameter of target lesions or the appearance of new lesions (Eisenhauer et al. 2009).

## Statistical methods

Data management and statistical analysis were by SPSS software version 13. Baseline laboratory markers were expressed as mean ± standard deviation (SD) or standard of error (SE) when appropriate. Progression-free survival and overall survival were analysed by the Kaplan–Meier method. Paired *t* test was used to compare AFP, size and number of focal lesions after therapy. ANOVA test was used when appropriate, p < 0.05 indicating statistically significance result.

## Results

The baseline characteristics of the patients under study were shown in Table 1. Four patients had one focal lesion (26.7 %), 3 patients had 2 focal lesions (20 %), 5 patients had 3 focal lesions (33.3 %), 3 patients had four focal lesions (20 %). 11 patients had portal vein thrombosis (73.3 %). The mean of the sum of tumoral size in its longitudinal diameter is 10.5 ± 4.1 cm, mean Child Turcotte Pugh score was 6.33 ± 0.3, MELD score 13.7 ± 1.4, PS <2.

**Table 1:** Baseline demographic, laboratory and radiological characteristics of the study patients and controls

Variable	SRB+CTB	SRB	Supportive	p
Age (years)	49.5 ± 5.2	47.2 ± 4.6	46.6 ± 4.3	0.2
BMI k/m^2^	27.7 ± 0.9	26.8 ± 0.5	27 ± 1.3	*0.03*
AST IU/l	97.6 ± 26.5	68 ± 15	89.5 ± 28.4	*0.005*
ALT IU/l	65 ± 12.2	85 ± 17	73.8 ± 11.5	*0.001*
Albumin gm/dl	3.37 ± 0.32	3.6 ± 0.3	3.4 ± 0.3	0.09
T.Bilirubin mg/dl	2.03 ± 0.3	1.94 ± 0.9	2.2 ± 0.4	0.48
WBC cell/µl	3.7 ± 0.3	4.3 ± 0.4	3.6 ± 0.5	*0.000*
HB gm/dl	11.2 ± 0.8	11.6 ± 0.6	10.9 ± 0.5	*0.02*
Platelets cell/µl	91.3 ± 8.3	100 ± 7	94.2 ± 10.2	*0.03*
INR	1.35 ± 0.08	1.32 ± 0.3	1.4 ± 0.1	0.5
Creatinine mg/dl	1.2 ± 0.5	0.9 ± 0.4	1.1 ± 0.3	0.14
CTP	6.3 ± 0.3	6.7 ± 0.7	6.5 ± 0.2	0.07
MELD	13.7 ± 1.4	14.2 ± 1.6	14 ± 1.2	0.6
AFP ng/dl (mean ± SE)	6211 ± 929	3154 ± 345	2710 ± 581	*0.000*
No of focal lesions	2.5 ± 1.1	2.4 ± 0.3	2.4 ± 0.9	0.9
Size (cm)	10.5 ± 4.1	11.2 ± 6.3	8.7 ± 2.4	0.3
PVT (n)	11(73.3 %)	12 (75 %)	9 (60 %)	0. 8
Survival (months)	12.9 ± 2.1	7.9 ± 0.9	4.5 ± 1.3	*0.000*

Italic values indicate significant differences when p < 0.05

*SRB* sorafenib, *CTB* capcitabine, *BMI* body mass index, *CTP* child turcott pugh, *MELD* model for end stage liver disease, *AFP* alfafetoprotein

The patients were given the planned medications and followed up after 1 month with AFP level, abdominal USG and triphasic CT after 3 months to detect the number and size of lesions. During therapy, 6 patients developed rising bilirubin (40 %) to grade 3 which improved after adding ursodeoxycholic acid, worsening thrombocytopenia in 4 patients (26.7 %) which improved after temporary cessation of therapy with adding vitamin B complex and eltrombobag (Revolade) 25 mg daily, melena in 2 patients (13.3 %) which were improved with temporary cessation of therapy and blood transfusion. Hand and foot syndrome in 3 patients (20 %) which was managed by temporary cessation of therapy, topical moisturizing and antibiotic creams and paracetamol for pain relief.

After *1* *month* of therapy as shown in Table 2; a highly significant reduction in the size of lesions and AFP level (p = 0.000) but the number of lesions is not changed.

**Table 2:** Tracing of the effects of combined therapy on AFP, number and size of focal lesions during the follow up period

	1 month	4th month	7th month	10th month	Basal
AFP	57 ± 17.5	6.9 ± 1.5	7.1 ± 1.1	21.3 ± 9.4	6211.3 ± 929
p	(0.000)	(0.000)	(0.6)	(0.4)	–
No of lesions	2.4 ± 1	1.47 ± 0.6	0.93 ± 0.4	0.73 ± 0.34	2.5 ± 1.1
P	(p = 0.8)	(p = 0.000)	(p = 0.001)	(p = 0.2)	–
Size of lesions	6.4 ± 2.9	3.8 ± 1.8	2.2 ± 1.4	2.2 ± 0.9	10.4 ± 4.1
p	(p = 0.000)	(p = 0.000)	(p = 0.001)	(p = 0.8)	–

*Three months* later, the patients were doing well with medications and showed a highly significant reduction of the number, size of the lesions with a further significant reduction in AFP level.

After further *3* *months* i.e. after 7 months of therapy; a further significant reduction in number and size of lesions (p = 0.001), APF reached a constant level with no significant change from the previous reading. 3 patients showed complete response with complete disappearance of the lesions (20 %).

*Three months* later (10 months of therapy); non-significant reduction in the number and size of lesions, another 3 patients showed complete response with the disappearance of focal lesions (20 %). AFP mean value unexpectedly was higher in one patient (6.7 %) due to recurrence of the disease with rising of AFP to 230 ng/ml.

After 10 months of therapy, 6 patients showed a complete response (40 %) and 2 of them showed complete recanalization of portal vein so treatment was stopped and the others (n = 4) showed partial portal vein recanalization. Patients who achieved complete response with partial recanalization of the portal vein or patients who achieved partial response i.e. reduction in number and size of focal lesions (n = 8, 53.3 %) were kept on therapy for 21 days and repeated every 14 days with continued follow up every 2 months. One patient (6.7 %) showed recurrence of the disease and died after 1 month. The medications were safe and tolerable by the patients in this low dose regimen.

The control group composed of 30 male patients with advanced HCC; they were divided into supportive treatment arm and sorafenib arm, their baseline demographic, laboratory and radiological characteristics were shown in Table 1. A statistically significant difference in BMI, AST, ALT, WBCs, hemoglobin, platelet count and AFP was present when compared to the study patients.

**Fig. 1:**
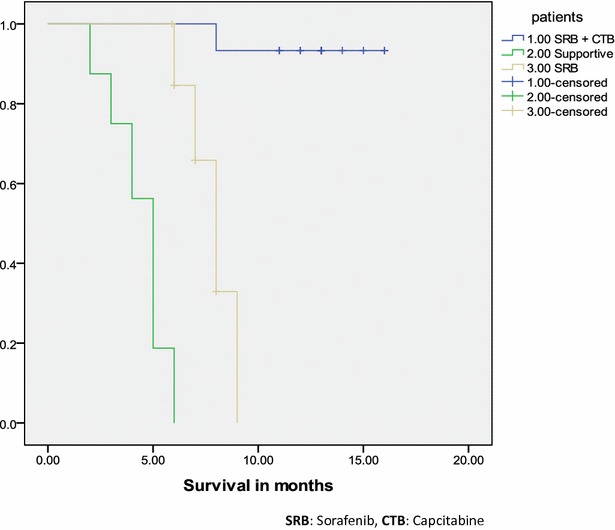
Kaplan Meier plot for survival in study and control groups

The control group showed a highly significant reduction in survival when compared to the patients who received capecitabine and sorafenib (p = 0.000) as shown in Table 1 and Fig. 1. It was proved that adding capecitabine was more superior to sorafenib alone or treatment naïve patients.

## Discussion

With the introduction of various radiological and laboratory modalities, still they did not provide sufficient sensitivity and specificity for early prediction of HCC nor helping patients so much as most of them present at the time of diagnosis in an inoperable stage with limited chance of curative therapy, so for the majority of patients only palliative therapy can be offered.

Administration of systemic chemotherapy to patients with advanced liver disease remains a challenging issue. Previous studies on capecitabine provided evidence on its relative safety and tolerance in patients with average functional hepatic reserve (Eklund et al. 2005).

A study conducted at 2004 reported the safe use of capecitabine for hepatobiliary cancer, with the occurrence of few side effects as palmoplantar erythrodysesthesia and mild hematologic toxicity (Patt et al. 2004).

Another study conducted at 2007 which stated that capecitabine could be safely given in patients with advanced HCC with compensated cirrhosis in an outpatient setting. One partial response was observed and 3-month progression free survival rate was 27 %. The median time to tumor progression and median overall survival were 2.2 months (95 % CI 1.7–2.7 months) and 10.1 months (95 % CI 3.0–17.2 months) (Von Delius et al. 2007).

In patients with Child-Pugh Class A, the toxicity of these agents occurs with the same frequency as in patients with normal hepatic function (Walko and Lindley 2005).

In comparison with doxorubicin; patients treated with capecitabine showed 6 months median progression-free survival, which was much longer than that reported about the former (Gish et al. 2007).

The SHARP (Lovet et al. 2008) and the Asia–Pacific (Cheng et al. 2009) trials revealed that sorafenib was associated with a statistically significant improved survival in advanced HCC with adequate hepatic functional reserve however; absolute advantage is unsatisfactory (2.8 months among Western patients; 2.3 months among Asians).

One of the most important issues is that the favorable effects of sorafenib on tumor vascularization and disease progression are rapidly lost after stoppage of treatment with the possibility of a rebound increase in tumor growth (Wolter et al. 2009), and short courses of treatment could change tumor into a more aggressive phenotype (Pàez-Ribes et al. 2009).

The antiangiogenic effect of chemotherapy can be augmented by continuous low-dose administration, and this low dose approach is characterized by good tolerability and low toxicity profile (Kerbel and Kamen 2004).

A report of 2 cases treated with metronomic capecitabine, they showed unexpectedly good therapeutic efficacy and the authors concluded that this treatment might be considered in patients unresponsive or intolerant to sorafenib, or when sorafenib is contraindicated (Marinelli et al. 2013).

Abdel-Rahman et al. (2013) concluded that in advanced HCC, capecitabine is inferior to sorafenib in terms of median progression-free survival and overall survival, and it should not be used alone but combination therapy with sorafenib should be considered.

Another case report of good response and long progression free survival of 50 months when capecitabine in low dose was used as a second line therapy in HCC resistant or unfit for sorafenib (Solda et al. 2015).

Granito et al. (2015) showed that disease control was achieved by metronomic capecitabine in (23 %) of patients. Median time-to-progression was 4 months. Median overall survival was 8 months (95 % confidence interval 3.7–12.3) and they concluded that metronomic capecitabine was well tolerated and exhibited a potential anti-tumour activity with long-lasting disease control.

In the current study conducted on 15 patients presented with advanced HCC who were not candidate for surgical or loco regional therapy and after their approval to be enrolled and treated with the defined therapy; they received capecitabine 500 mg per day combined with sorafenib 200 mg twice daily till normalization of AFP then the treatment was modified to give capecitabine 250 mg every other day and sorafenib 400 mg once daily until complete response, progression of disease, unacceptable toxicity.

After 10 months of therapy; 6 patients showed a complete response (40 %). One patient (6.7 %) showed recurrence of the disease and died after 1 month, 8 patients showed partial response (53.3 %). Patients who were kept on medications till now showed accepted safety and tolerability with proven highly significant prolongation of survival when compared to sorafenib only and treatment naïve controls (12.9 ± 2.1, 7.9 ± 0.9, 4.5 ± 1.3 months respectively, p = 0.000).

Results are consistent with of Brandi et al. as regard overall survival however the current study showed a higher number of patients with complete response; their study enrolled 59 patients treated with metronomic capecitabine; median progression-free survival of 6.03 months and an overall survival of 14.47 months. Two patients achieved a complete response, 1 patient achieved partial response (Brandi et al. 2013).

Patients who showed complete response with complete recanalization of the portal vein achieved the end of treatment and stopped therapy with continued follow up every 2 months.

From data revealed by the current study, combined low dose capecitabine and sorafenib proved to be safe, enhances the effects of sorafenib, with low toxicity profile and deserves further attention as a convenient, outpatient-based chemotherapy regimen in patients with advanced HCC and adequate hepatic reserve.

## Abbreviations

AFP: alfa fetoprotein
BCLC: Barcelona Clinic Liver Cancer
CT: computerized tomography
CTP: Child Turcot Pugh
DEBs: drug-eluting beads
HB: hemoglobin
HCC: hepatocellular carcinoma
HCV: hepatitis C virus
MELD: model for end stage liver disease
PDGFR: platelet endothelial growth factor receptors
PVT: portal vein thrombosis
PS: performance status
Raf: rapidly accelerated fibrosarcoma
RCTs: randomized controlled trials
SD: standard deviation
SE: standard of error
SPSS: statistical package for social science
TACE: transcatheter arterial chemoembolization
USG: ultrasonography
VEGFR: vascular endothelial growth factor receptors
